# Multifunctional two-stage riser fluid catalytic cracking process

**DOI:** 10.1007/s13203-014-0079-5

**Published:** 2014-09-03

**Authors:** Jinhong Zhang, Honghong Shan, Xiaobo Chen, Chunyi Li, Chaohe Yang

**Affiliations:** State Key Laboratory of Heavy Oil Processing, College of Chemical Engineering, China University of Petroleum, Qingdao, 266580 China

**Keywords:** Fluid catalytic cracking, Two-stage riser, Gasoline upgrading, Diesel, Heavy oil

## Abstract

This paper described the discovering process of some shortcomings of the conventional fluid catalytic cracking (FCC) process and the proposed two-stage riser (TSR) FCC process for decreasing dry gas and coke yields and increasing light oil yield, which has been successfully applied in 12 industrial units. Furthermore, the multifunctional two-stage riser (MFT) FCC process proposed on the basis of the TSR FCC process was described, which were carried out by the optimization of reaction conditions for fresh feedstock and cycle oil catalytic cracking, respectively, by the coupling of cycle oil cracking and light FCC naphtha upgrading processes in the second-stage riser, and the specially designed reactor for further reducing the olefin content of gasoline. The pilot test showed that it can further improve the product quality, increase the diesel yield, and enhance the conversion of heavy oil.

## Introduction

Fluid catalytic cracking (FCC) was an important process for converting heavy oil into high octane gasoline, diesel and liquified petroleum gas (LPG). In China, about 75–80 % of gasoline and 30–40 % of diesel comes from FCC products [1, 2]. Currently, approximately one-third of the world’s propylene is provided by the FCC modified processes [3].

As the crude oils are getting heavier and the demand for high value petroleum products is increasing, the FCC technologies are developing under the following directions: (a) reducing the yields of dry gas and coke, and increasing the yield of light oil; (b) increasing the diesel to gasoline ratio of the product; (c) increasing the processing capacity of inferior feedstocks; (d) improving the product quality; and (e) increasing the yield of light olefins. Thus, the two-stage riser (TSR) FCC process was developed, which can be operated in different modes for market demands. This paper will describe the TSR FCC process for maximizing light oil and its development for increasing diesel yield, enhancing the conversion of heavy oil, and reducing the olefin content of gasoline. The TSR FCC process for increasing light olefin yield will be described in another paper.

## TSR FCC process for maximizing light oil

In the conventional FCC process, the preheated high-boiling petroleum feedstock consisting of long-chain hydrocarbon molecules is mixed with cycle oil from the bottom of the distillation column and injected into the bottom of riser reactor where it is vaporized and cracked into smaller molecules by contacting with the hot regenerated catalyst from the regenerator. All of the cracking reactions take place in approximately 3 s. The coked catalyst and oil vapor are separated through a set of two-stage cyclones, then the coked catalyst is sent to the regenerator after stripping, and the oil vapor is piped to the fractionator.

Since the start-up of the first commercial FCC unit in 1942 [4], many improvements have been made in the field of catalysts, feed nozzles and rapid gas–solid separation equipment. However, the problem existed in the heart of the FCC unit—riser reactor is still not solved, which causes low yield of light oil, high yields of dry gas and coke, and poor quality of the FCC diesel.

**Fig. 1:**
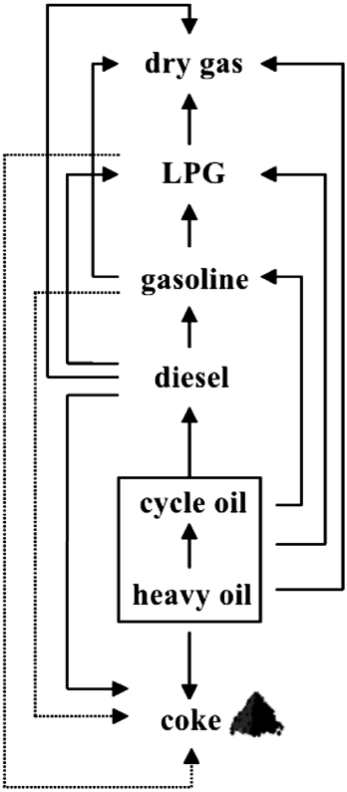
Parallel-sequential reaction network of heavy oil catalytic cracking

In order to solve these problems, the reaction mechanism of heavy oil in the riser reactor was studied firstly. Chemical reactions in the heavy oil catalytic cracking process are very complex. The heavy oil with complicated chemical structures and compositions crack into multiple products, which are also very complicated in structures and compositions, by a large set of parallel-sequential reactions (Fig. 1). The reactions proceed fast coupled with catalyst deactivation, and catalytic cracking happened simultaneously with thermal cracking. As can be seen in Fig. 1, if we want to increase the yields of light oil (gasoline and diesel) and LPG, the disadvantaged secondary reactions (such as the overcracking of the desired products, dehydrogenation and condensation reactions) should be inhibited, and the yields of the ultimate products (dry gas and coke) should be reduced.

To understand the essence of heavy oil catalytic cracking process, the online sampling device was developed [5], and the gas–liquid–solid three phase online sampling of high-temperature industrial riser reactor was realized for the first time [6]. Based on the analysis of products and catalysts obtained from different axial positions, a new method for investigating the reaction process in industrial riser reactor was developed.

Research found that the conventional RFCC reactor has some shortcomings, such as the overlong reaction time, the low overall activity and selectivity of the catalyst, and the competitive adsorption and reaction between fresh feedstock and cycle oil [6].

1. Overlong reaction time will lead to the overcracking of light oil (gasoline and diesel). Figure 2 illustrates the simulated results of product distribution along an industrial riser reactor. It can be seen, the gasoline yield increased rapidly in the first 10 m above the feed inlet, then rose slightly and finally reached a plateau. By contrast, the maximum diesel yield was obtained at about 5 m above the feed inlet, and the yield of light oil achieved the maximum level at around 10 m above the feed inlet. As can be seen, gasoline and diesel are mainly generated at the feed entrance zone of the riser reactor (about 1 s of residence time) [7], afterwards, the overcracking of light oil happens, and the yield of dry gas and coke increased. Thus, the reaction time in the conventional riser reactor should be shortened.

**Fig. 2:**
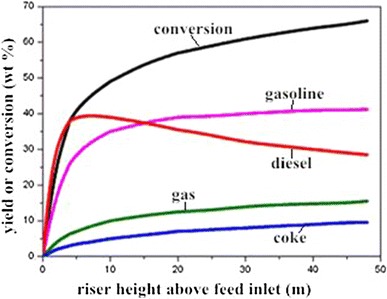
Product yield as a function of riser height
2. The overall activity and selectivity of the catalyst in the conventional riser reactor are seriously insufficient. Based on the online sampling study, axial variation of catalyst activity along the riser reactor was obtained, which indicated that the activity of catalyst rapidly decreased to 40–50 % of the initial level in the feeding zone. The significant decrease of catalyst activity and selectivity leads to aggravated thermal cracking reactions and reduced selectivities of desired products in the second half of the riser reactor. Thus, increasing the catalyst activity, especially in the second half of the riser reactor, is a key matter for developing novel FCC process.
3. The inhibiting effect of competitive adsorption between fresh and cycle oil on desirable reactions aggravates the product distribution. Fresh feedstock contains a considerable amount of high-boiling-point heavy components with large molecular weight, which are easy to crack. However, it is difficult for these components to vaporize, diffuse and adsorb on the active sites. By contrast, the cycle oil contains a large amount of aromatics, which are difficult to crack. But the cycle oil has a narrower boiling range, thus it is easier to vaporize and diffuse to grab the active sites, and influence the adsorption and reaction of the fresh feedstock. Figure 3 compares the weighted results of separated cracking of fresh feed and cycle oil with the consequences of the mixed feeding scheme. It can be found that, at the similar conversion level, when fresh feed and cycle oil were fed and cracked separately, the product distribution significantly improved, lower yields of dry gas and coke, and higher yields of liquid products (LPG + gasoline + diesel) can be achieved.

**Fig. 3:**
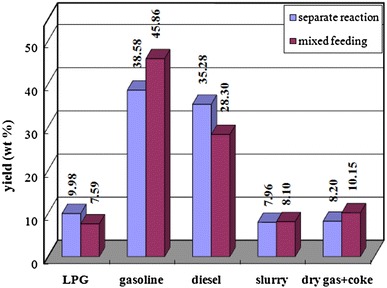
Effect of separate reaction on the product distribution

On the basis of above analysis, we designed two structure-optimized riser reactors to replace the conventional riser reactor [8], formed a novel reaction-regeneration system with two catalyst circulation routes, successfully realized it in commercial scale and finally developed the two-stage riser FCC technology.

**Fig. 4:**
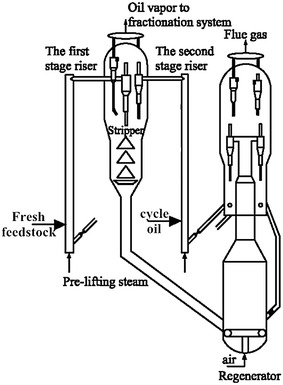
Schematic diagram of the TSR FCC process

Figure 4 shows the schematic diagram of the reaction-regeneration system of the TSR FCC process. The fresh feed after heat-exchanging (preheating) is injected from the bottom of the first stage riser, then contacts with the hot regenerated catalyst, resulting in rapid vaporization and reaction. After about 1.0 s, the oil vapor and coked catalyst are separated. The coked catalyst was stripped by steam, then transported to the regenerator for regeneration. The oil vapor is piped to the fractionator and separated into different fractions, such as dry gas, LPG, gasoline, diesel (or LCO) and heavy cycle oil. The cycle oil is recycled to the bottom of the second stage riser and reacts over the regenerated catalyst (1.0–2.0 s). The oil vapor and coked catalyst are separated at the riser exit. The coke catalyst is regenerated after steam stripping, and the oil vapor is piped to the fractionator with that from the first riser. Thus, both the fresh feed and cycle oil can react over regenerated catalyst with high activity and under the most favorable conditions for maximum light oil yield, respectively.

In 2002, the TSR FCC technology was firstly applied in a 100 kt/a industrial unit belonging to the Shtar Science and Technology Group. The conventional riser reactor (45.0 m) was replaced by a 16.0 m riser reactor for the first stage reaction and a 10.7 m riser reactor for the second stage reaction. After the technological renovation and transformation, the dry gas and coke yields decreased 2.7 wt %, while the liquid products yield increased 2.7 wt %. Moreover, the cetane number of diesel increased 7 U.

At present, there are 12 industrial units, including that in the stage of transformation or new construction, applied the TSR technology. The accumulative processing capacity has reached 9 Mt/a, the processing capacity of the largest unit is 1.6 Mt/a.

## Multifunctional two-stage riser FCC process

The TSR technology is a great progress of FCC technology. However, as the crudes are getting heavier, the standard of gasoline becomes increasingly strict, and the greater demand of diesel, FCC units are facing many new challenges. Thus, the multifunctional two-stage riser (MFT) FCC process was proposed aimed to enhance the conversion of inferior feedstocks, upgrade FCC gasoline (reduce the olefin content) under minimum loss, and increase the diesel yield [9].

**Fig. 5:**
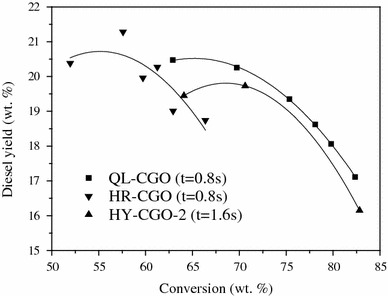
Relationship of conversion and the diesel yield [9]

The key problem for increasing diesel yield is that the maximal diesel yield always exists under moderate operating conditions while the feed conversion is very low (Fig. 5). This is because that catalytic cracking is a parallel-sequential reaction [10] and the diesel fraction is more susceptible to sequential cracking reactions than gasoline fractions [11]. In the conventional FCC process, the contradiction of increasing diesel yield and feed conversion is difficult to be resolved, especially when processing inferior feedstocks. The two-stage riser FCC process can solve this problem, using the first stage riser to produce more diesel, but this would lead to the increase of HCO yield. Thus, measures should be taken to enhance the conversion of HCO. As the crackability of HCO is poorer than the fresh feed, higher operating severity (higher reaction temperature and catalyst-to-oil ratio) should be taken to enhance the conversion of HCO.

One of the major improvements of the MFT FCC process is to efficiently upgrade the FCC gasoline, reducing the olefin content under minimum loss. Four measures were carried out in the MFT process.

**Fig. 6:**
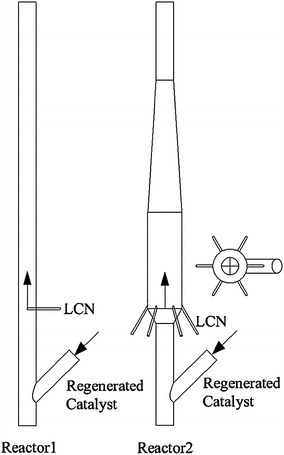
Schematic diagram of the conventional and the novel riser reactor [21]

1. Recycling the light naphtha selectively. This is because gasoline olefins with a carbon number of seven or higher are easy to crack, while pentene and hexene are difficult to crack under conventional FCC operating conditions [12–15]. Moreover, light naphtha has higher olefin content, which is helpful to restrain the protolytic cracking [16].
2. Decreasing the temperature difference between naphtha and regenerated catalysts. Research found that this operation could restrain the thermal cracking and improve product distribution [17]. It can be conducted by cooling the regenerated catalyst and pre-heating the naphtha feed.
3. Using the partial-coked catalyst to upgrade naphtha. Corma et al. [18] found that the Y zeolite with a coke-on-catalyst content of 1.2 wt % still has enough activity for transforming olefins into paraffins through hydrogen-transfer reaction. Zhang et al. [19] and Yuan et al. [20] found that little coke on catalyst could reduce the dry gas and coke yields and increase the gasoline yield. Thus, this measure is helpful to reduce the naphtha loss.
4. Optimizing the structure of the reactor. Wang et al. [22] and Lu et al. [23] studied the gas-solids flow patterns in the diameter-enlarged reactor by cold model and computational fluid dynamics (CFD) simulation and found that the solid density at the bottom of the enlarged section significantly increased, which is beneficial for bimolecular hydrogen transfer reactions. In the previous work [21], we designed a novel structurally changed reactor with a multinozzle feed system (Fig. 6). Experimental results show that significantly increased olefin conversion and reforming efficiency, as well as improved hydrogen utilization rate can be achieved. Therefore, optimizing the structure of the reactor is also very important.

**Fig. 7:**
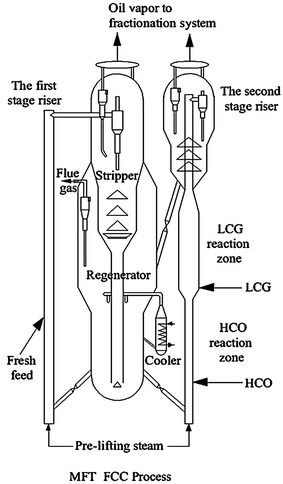
Schematic diagram of the MFT FCC process [9]

The schematic diagram of the MFT process is shown in Fig. 7. The fresh feed reacts at moderate operating conditions in the first stage riser to generate more diesel oil. Then the oil gas is piped to the fractionator and separated into dry gas, LPG, light cracking gasoline (LCG), heavy cracking gasoline (HCG), diesel and heavy cycle oil (HCO). The HCO is recycled to the bottom of the second stage riser while the LCG is injected from the bottom of the enlarged section. As the injection of LCG, more heat should be provided by the catalyst. According to the heat balance, the catalyst circulation should be enlarged. Thus, the HCO could be converted at a higher severity without raising the riser outlet temperature, and the LCG could be upgraded at a lower severity over temperature-lowered and partially-coked catalyst. The final gasoline product is the mixture of the HCG from the first fractionator and the full-range gasoline from the second fractionator. It should be noted that this process is designed for experimental research, because in the pilot-scale unit, the two stage riser cannot run simultaneously. If in commercial units, the two risers can share a disengager and fractionator.

Simulated experiments were carried out in a pilot scale riser FCC apparatus with HY-CGO as the feedstock and a Y-zeolite based equilibrium FCC catalyst. The results show that, comparing with the TSR FCC process, the MFT FCC process increased feed conversion and diesel yield with 2.2 and 3.3 wt %, respectively, as well as the olefin content of gasoline decreased approximately 17 wt %. Moreover, the cetane number of diesel can be increased. However, the dry gas and coke yields also increased about 1.3 wt %. Thus, it needs to be further improved.

## Conclusion

In the conventional RFCC reactor, the overlong reaction time, the fast deactivation of catalyst, and the competitive adsorption and reaction between fresh feedstock and cycle oil would lead to the overcracking of light oil and the deterioration of the product distribution. The proposed two-stage riser FCC process successfully solved these problems, which can decrease the dry gas and coke yields, increase the light oil yield and improve the product quality. To adapt to the new challenges, the multifunctional two-stage riser FCC process was proposed, which can enhance the conversion of heavy oil, increase the diesel yield and further improve the product quality, especially reduce the olefin content of gasoline.
